# Bis[3,5-di­fluoro-2-(pyridin-2-yl)phen­yl](4,4′-di­meth­oxy-2,2′-bi­pyridine)­iridium(III) hexa­fluorido­phosphate

**DOI:** 10.1107/S2414314622008306

**Published:** 2022-08-26

**Authors:** Madelyn R. Shevlin, Emily E. Stumbo, Colin D. McMillen, Jared A. Pienkos

**Affiliations:** a Department of Chemistry and Physics, University of Tennessee at Chattanooga, Chattanooga, TN 37403, USA; b Department of Chemistry, Clemson University, Clemson, SC 29634, USA; Purdue University, USA

**Keywords:** crystal structure, 4–4′-dimeth­oxy-2,2′-bi­pyridine, iridium(III), cyclo­metalated complex

## Abstract

The title cyclo­metalated distorted octa­hedral iridium complex, [Ir(C_11_H_6_F_2_N)_2_(C_12_H_12_N_2_O_2_)]PF_6_, exhibits elongated Ir—N bonds to the dimeth­oxy bi­pyridine ligand [2.128 (3) and 2.136 (3) Å] where these nitro­gen atoms are *trans* to the Ir—C bonds of the two cyclo­metalating di­fluoro­phenyl­pyridine ligands. The angles between the mean planes of the phenyl and pyridyl fragments within the individual ligands range from 3.5 (2) to 11.4 (2)° to deviate slightly from coplanarity.

## Abstract 2

The title compound, bis­[3,5-di­fluoro-2-(pyridin-2-yl)phen­yl](4,4′-dimeth­oxy-2,2′-bi­pyridine)­iridium(III) hexa­fluorido­phosphate, is a distorted octa­hedral cyclo­metalated complex that exhibits a *trans* effect.

## Graphical abstract

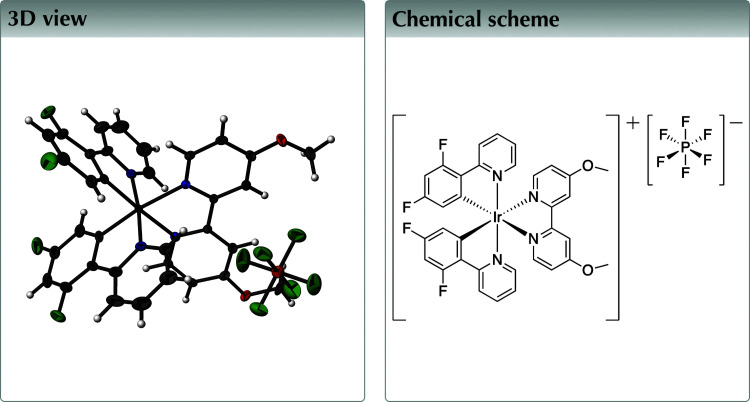

## Structure description

**Figure 1:**
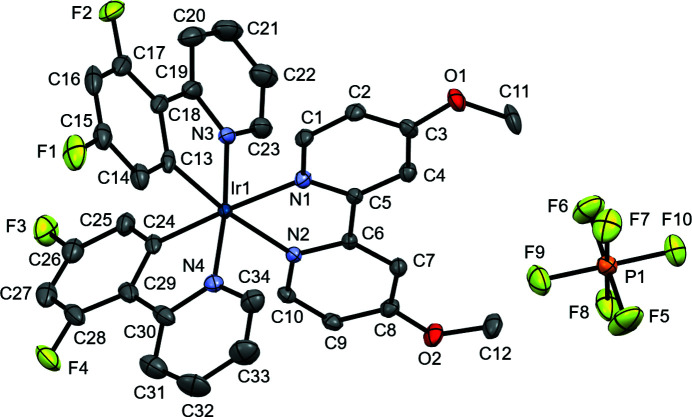
The structures of the molecular components of the title compound shown as 50% probability ellipsoids. Hydrogen atoms have been omitted for clarity.

The title compound, [Ir(dfppy)_2_(bipyOMe)](PF_6_), is a distorted octa­hedral complex composed of bidentate cyclo­metallated ligands. The photophysical properties of cyclo­metalated iridium(III) complexes have been studied extensively in diverse applications such as cell imaging and OLEDs (Lee *et al.*, 2009 ▸; Thorp-Greenwood, 2012 ▸; You *et al.*, 2014 ▸). In [Ir(dfppy)_2_(bipyOMe)](PF_6_), the nitro­gen atoms of the bipyOMe ligand are oriented *trans* to the carbon atoms of the dfppy ligands (Fig. 1 ▸). The Ir—N bond lengths to the bipyOMe ligand are 2.128 (3) and 2.136 (3) Å, longer than the Ir—N [2.035 (3) and 2.042 (3) Å] or Ir—C [2.014 (3) and 2.017 (3) Å] bonds to the dfppy ligands. This is consistent with a substantial *trans* effect directed by the carbon atoms of dfppy. This feature is also present in related structures in the literature, including for example Ir—N bonds to the *trans*-effected nitro­gen atoms of the hydrogen pyridin-2-yl-phosophonato ligand of bis­[3,5-di­fluoro-2-(pyridin-2-yl)phen­yl](hydrogen pyridin-2-yl-phospho­nato)iridium(III) [Ir—N = 2.153 (4) Å; Zeng *et al.*, 2019 ▸], and of the bi­pyridine ligands in the structures of (2,2′-bi­pyridine)­bis­[3,5-di­fluoro-2-(pyridin-2-yl)phen­yl]iridium(III) com­plexes [ranging from 2.120 (4) to 2.141 (4) Å; Li *et al.*, 2017 ▸; Moriuchi *et al.*, 2012 ▸]. This arrangement maximizes the number of C—Ir—N inter­ligand *trans*-inter­actions compared to other potential isomers that would orient only one of the bipyOMe nitro­gen atoms across from a strong *trans*-donor carbon atom, or that would have both strong *trans*-donor carbon atoms opposing one another (*i.e.* all nitro­gen atoms are *trans* to one another).

**Figure 2:**
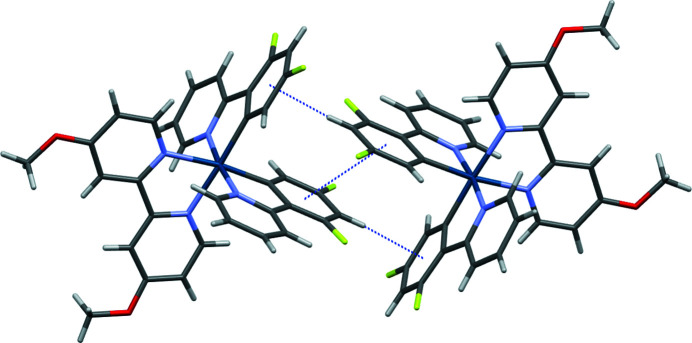
Dimers of the title complex formed *via* offset π stacking and C—H⋯π inter­actions. The projection is viewed along [010].

**Figure 3:**
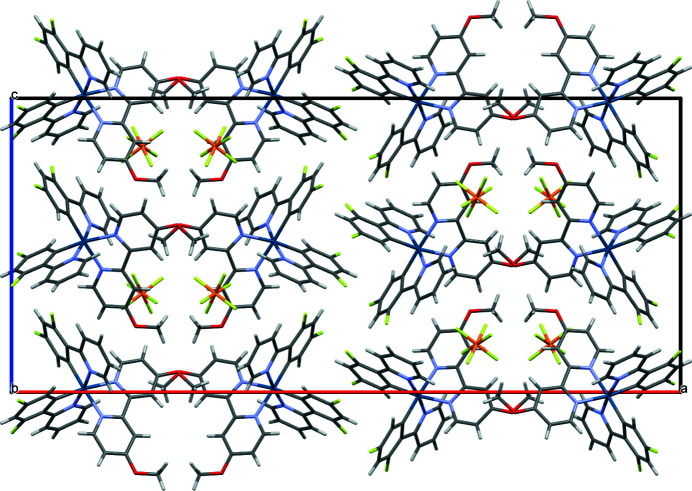
Packing diagram of the title compound viewed along [010].

In addition to the octa­hedral distortion arising from the *trans*-effect, an angular distortion occurs from the chelating ligands, with *cis*-angles about iridium ranging from 76.39 (10) to 101.08 (12)°, and *trans*-angles about iridium ranging from 172.49 (12) to 177.11 (12)°. The ligands themselves deviate slightly from coplanarity, with mean plane to mean plane angles between the phenyl and pyridyl fragments within the individual ligands ranging from 3.5 (2) to 11.4 (2)°. The methyl groups of the bipyOMe ligands both fold inward. Neighboring complexes form dimers (Fig. 2 ▸) through inter­actions between their dfppy ligands, including offset π stacking [centroid–centroid = 3.616 (3) Å; plane-to-plane distance = 3.202 (3) Å] and C—H⋯π (H⋯centroid = 2.52 Å) inter­actions. Several C—H⋯F inter­actions between the complexes and the (PF_6_)^−^ anions (Table 1 ▸, Fig. 3 ▸), as well as a C—H⋯O inter­action between meth­oxy groups of neighboring complexes further support the long-range packing.

## Synthesis and crystallization

[Ir(dfppy)_2_Cl]_2_ was prepared according to the literature (Skórka *et al.*, 2016 ▸). [Ir (dfppy)_2_Cl]_2_ (0.0508 g, 0.0418 mmol) and 4,4′-dimeth­oxy-2,2′-bi­pyridine (0.0208 g, 0.0962 mmol) were combined in ethyl­ene glycol (5 ml). The resulting yellow–green heterogenous mixture was heated under N_2_ to 150°C while stirring_._ After 20 h, the resulting yellow homogenous solution was allowed to cool to room temperature and 10 ml of NH_4_PF_6_ (sat. aq.) were added. The yellow precipitate that formed was collected by vacuum filtration, washed with H_2_O (3 × 10 ml), Et_2_O (3 ×10 ml), and dried *in vacuo* to give a yellow powder (0.0517 g, 78.3%). Yellow needle-like crystals suitable for X-ray diffraction were obtained by vapor–vapor diffusion from a solution of hexa­nes and di­chloro­methane. ^1^H NMR (400 MHz, DMSO-*d*_6_) δ 8.48 (*d*, *J* = 2.7 Hz, 2H), 8.28 (*d*, *J* = 8.5 Hz, 2H), 8.03 (*t*, *J* = 7.9 Hz, 2H), 7.75 (*dd*, 2H), 7.65 (*d*, *J* = 6.4 Hz, 2H), 7.28 (*td*, 4H), 6.97 (*d*, *J* = 11.8 Hz, 2H), 5.61 (*dd*, 2H), 4.00 (*s*, 6H).

## Refinement

Crystal data, data collection, and structure refinement details are summzarized in Table 2 ▸.

## Supplementary Material

Crystal structure: contains datablock(s) I. DOI: 10.1107/S2414314622008306/zl4051sup1.cif

Structure factors: contains datablock(s) I. DOI: 10.1107/S2414314622008306/zl4051Isup2.hkl

Supporting information file. DOI: 10.1107/S2414314622008306/zl4051Isup3.cdx

CCDC reference: 2202472

Additional supporting information:  crystallographic information; 3D view; checkCIF report

## Figures and Tables

**Table 1:** Hydrogen-bond geometry (Å, °)

*D*—H⋯*A*	*D*—H	H⋯*A*	*D*⋯*A*	*D*—H⋯*A*
C11—H11*A*⋯F7^i^	0.98	2.51	3.240 (6)	131
C11—H11*B*⋯O2^ii^	0.98	2.52	3.294 (5)	136
C20—H20⋯F2	0.95	2.25	2.866 (6)	122
C31—H31⋯F4	0.95	2.25	2.869 (6)	122
C34—H34⋯F8^iii^	0.95	2.55	3.412 (5)	150

Symmetry codes: (i) 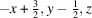 ; (ii) 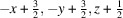 ; (iii) 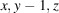 .

**Table 2:** Experimental details

Crystal data	
Chemical formula	[Ir(C_11_H_6_F_2_N)_2_(C_12_H_12_N_2_O_2_)]PF_6_
*M*_r_	933.74
Crystal system, space group	Orthorhombic, *P* *b* *c* *n*
Temperature (K)	100
*a*, *b*, *c* (Å)	41.574 (3), 8.6065 (7), 18.2384 (14)
*V* (Å^3^)	6525.9 (9)
*Z*	8
Radiation type	Cu *K*α
μ (mm^−1^)	9.27
Crystal size (mm)	0.20 × 0.07 × 0.06

Data collection	
Diffractometer	Bruker D8 Venture Photon 2
Absorption correction	Multi-scan (*SADABS*; Krause *et al.*, 2015 ▸)
*T*_min_, *T*_max_	0.667, 1.000
No. of measured, independent and observed [*I* > 2σ(*I*)] reflections	51121, 6215, 5595
*R*_int_	0.043
(sin θ/λ)_max_ (Å^−1^)	0.610

Refinement	
*R*[*F* ^2^ > 2σ(*F* ^2^)], *wR*(*F* ^2^), *S*	0.028, 0.068, 1.16
No. of reflections	6215
No. of parameters	471
H-atom treatment	H-atom parameters constrained
Δρ_max_, Δρ_min_ (e Å^−3^)	0.91, −0.61

Computer programs: *APEX3* (Bruker, 2017 ▸), *SAINT* (Bruker, 2016 ▸), *SHELXT2014/5* (Sheldrick, 2015*a* ▸), *SHELXL2016/6* (Sheldrick, 2015*b* ▸), and *Mercury* (Macrae *et al.*, 2020 ▸), *publCIF* (Westrip, 2010 ▸).

## full crystallographic data

### Crystal data

**Table:**

[Ir(C_11_H_6_F_2_N)_2_(C_12_H_12_N_2_O_2_)]PF_6_	*D*_x_ = 1.901 Mg m^−^^3^
*M**_r_* = 933.74	Cu *K*α radiation, λ = 1.54178 Å
Orthorhombic, *P**b**c**n*	Cell parameters from 9410 reflections
*a* = 41.574 (3) Å	θ = 4.0–70.2°
*b* = 8.6065 (7) Å	µ = 9.27 mm^−^^1^
*c* = 18.2384 (14) Å	*T* = 100 K
*V* = 6525.9 (9) Å^3^	Column, yellow
*Z* = 8	0.20 × 0.07 × 0.06 mm
*F*(000) = 3632	

### Data collection

**Table:**

Bruker D8 Venture Photon 2 diffractometer	5595 reflections with *I* > 2σ(*I*)
Radiation source: Incoatec IµS	*R*_int_ = 0.043
φ and ω scans	θ_max_ = 70.3°, θ_min_ = 5.0°
Absorption correction: multi-scan (SADABS; Krause *et al.*, 2015)	*h* = −50→50
*T*_min_ = 0.667, *T*_max_ = 1.000	*k* = −9→10
51121 measured reflections	*l* = −20→22
6215 independent reflections	

### Refinement

**Table:**

Refinement on *F*^2^	Primary atom site location: dual
Least-squares matrix: full	Secondary atom site location: difference Fourier map
*R*[*F*^2^ > 2σ(*F*^2^)] = 0.028	Hydrogen site location: inferred from neighbouring sites
*wR*(*F*^2^) = 0.068	H-atom parameters constrained
*S* = 1.16	*w* = 1/[σ^2^(*F*_o_^2^) + (0.0194*P*)^2^ + 21.8494*P*] where *P* = (*F*_o_^2^ + 2*F*_c_^2^)/3
6215 reflections	(Δ/σ)_max_ = 0.008
471 parameters	Δρ_max_ = 0.91 e Å^−^^3^
0 restraints	Δρ_min_ = −0.61 e Å^−^^3^

### Special details

**Table:**

Geometry. All esds (except the esd in the dihedral angle between two l.s. planes) are estimated using the full covariance matrix. The cell esds are taken into account individually in the estimation of esds in distances, angles and torsion angles; correlations between esds in cell parameters are only used when they are defined by crystal symmetry. An approximate (isotropic) treatment of cell esds is used for estimating esds involving l.s. planes.
Refinement. H atoms were placed in calculated positions with C—H bond distances of 0.95 Å for aromatic 0.98 Å for CH_3_ moieties, respectively. Methyl H atoms were allowed to rotate but not to tip to best fit the experimental electron density. *U*_iso_(H) values were set to a 1.5 (for CH_3_) or 1.2 (for C—H) times *U*_eq_(C).

### Fractional atomic coordinates and isotropic or equivalent isotropic displacement parameters (Å^2^)

**Table:**

	*x*	*y*	*z*	*U*_iso_*/*U*_eq_	
Ir1	0.60823 (2)	0.58537 (2)	0.48740 (2)	0.01420 (6)	
P1	0.69527 (2)	1.06167 (12)	0.66335 (5)	0.0244 (2)	
F1	0.51080 (6)	0.1773 (4)	0.52914 (15)	0.0471 (7)	
F2	0.50104 (6)	0.6651 (4)	0.63715 (13)	0.0475 (7)	
F3	0.53664 (7)	0.9251 (3)	0.29752 (15)	0.0457 (7)	
F4	0.56046 (7)	0.4119 (3)	0.23471 (13)	0.0439 (7)	
F5	0.67444 (7)	0.9209 (3)	0.6335 (2)	0.0588 (9)	
F6	0.71604 (7)	1.2005 (3)	0.69438 (17)	0.0487 (7)	
F7	0.70871 (7)	0.9525 (3)	0.72743 (16)	0.0484 (7)	
F8	0.68154 (6)	1.1714 (4)	0.60026 (14)	0.0473 (7)	
F9	0.72483 (6)	1.0121 (4)	0.61233 (14)	0.0477 (7)	
F10	0.66573 (6)	1.1117 (3)	0.71486 (14)	0.0392 (6)	
O1	0.68565 (6)	0.4864 (3)	0.77561 (13)	0.0252 (6)	
O2	0.74782 (6)	0.8299 (3)	0.43096 (14)	0.0276 (6)	
N1	0.63528 (7)	0.5248 (3)	0.58221 (15)	0.0172 (6)	
N2	0.65522 (6)	0.6726 (3)	0.46407 (15)	0.0153 (5)	
N3	0.59314 (7)	0.7760 (4)	0.54453 (15)	0.0211 (6)	
N4	0.61822 (7)	0.3919 (4)	0.42744 (16)	0.0207 (6)	
C1	0.62284 (8)	0.4565 (4)	0.64235 (19)	0.0204 (7)	
H1	0.601441	0.418006	0.640197	0.024*	
C2	0.63967 (8)	0.4401 (4)	0.70643 (19)	0.0215 (7)	
H2	0.630255	0.389275	0.747379	0.026*	
C3	0.67100 (8)	0.4995 (4)	0.71064 (18)	0.0184 (7)	
C4	0.68430 (8)	0.5666 (4)	0.64852 (19)	0.0186 (7)	
H4	0.705778	0.604258	0.649117	0.022*	
C5	0.66579 (8)	0.5780 (4)	0.58555 (18)	0.0152 (6)	
C6	0.67759 (8)	0.6531 (4)	0.51767 (17)	0.0159 (6)	
C7	0.70924 (8)	0.7019 (4)	0.50924 (18)	0.0189 (7)	
H7	0.724542	0.684919	0.547080	0.023*	
C8	0.71818 (8)	0.7759 (4)	0.44450 (19)	0.0205 (7)	
C9	0.69516 (9)	0.7961 (4)	0.38958 (18)	0.0210 (7)	
H9	0.700598	0.846105	0.344825	0.025*	
C10	0.66460 (8)	0.7424 (4)	0.40150 (17)	0.0177 (7)	
H10	0.649158	0.754997	0.363565	0.021*	
C11	0.71886 (10)	0.5355 (6)	0.7799 (2)	0.0352 (10)	
H11A	0.731560	0.479276	0.743261	0.053*	
H11B	0.727276	0.512989	0.828998	0.053*	
H11C	0.720241	0.647362	0.770505	0.053*	
C12	0.77258 (9)	0.7963 (5)	0.4834 (2)	0.0307 (9)	
H12A	0.767154	0.844654	0.530472	0.046*	
H12B	0.793125	0.837818	0.465787	0.046*	
H12C	0.774373	0.683614	0.489807	0.046*	
C13	0.56560 (8)	0.5034 (5)	0.52336 (18)	0.0237 (8)	
C14	0.55276 (10)	0.3574 (5)	0.5117 (2)	0.0300 (9)	
H14	0.564116	0.283327	0.483050	0.036*	
C15	0.52304 (9)	0.3197 (6)	0.5421 (2)	0.0332 (9)	
C16	0.50576 (10)	0.4217 (6)	0.5846 (2)	0.0381 (11)	
H16	0.485645	0.393021	0.605269	0.046*	
C17	0.51855 (10)	0.5655 (6)	0.5960 (2)	0.0360 (11)	
C18	0.54814 (9)	0.6118 (5)	0.5669 (2)	0.0279 (9)	
C19	0.56351 (9)	0.7625 (5)	0.5786 (2)	0.0290 (9)	
C20	0.55180 (11)	0.8876 (6)	0.6201 (2)	0.0403 (11)	
H20	0.531358	0.880956	0.643223	0.048*	
C21	0.57016 (12)	1.0204 (6)	0.6272 (2)	0.0426 (11)	
H21	0.562063	1.106271	0.654293	0.051*	
C22	0.60004 (11)	1.0293 (6)	0.5955 (2)	0.0374 (10)	
H22	0.613287	1.118091	0.602228	0.045*	
C23	0.61029 (9)	0.9055 (5)	0.5534 (2)	0.0273 (8)	
H23	0.630572	0.912941	0.529681	0.033*	
C24	0.58468 (8)	0.6421 (5)	0.39470 (18)	0.0222 (7)	
C25	0.56931 (9)	0.7793 (5)	0.3780 (2)	0.0285 (8)	
H25	0.570615	0.865422	0.410381	0.034*	
C26	0.55177 (9)	0.7906 (6)	0.3128 (2)	0.0336 (9)	
C27	0.54847 (9)	0.6700 (6)	0.2651 (2)	0.0340 (10)	
H27	0.535858	0.679906	0.221947	0.041*	
C28	0.56399 (10)	0.5335 (6)	0.2816 (2)	0.0328 (9)	
C29	0.58303 (9)	0.5150 (5)	0.34384 (19)	0.0273 (8)	
C30	0.60312 (10)	0.3802 (5)	0.3612 (2)	0.0269 (8)	
C31	0.60924 (11)	0.2514 (6)	0.3162 (2)	0.0384 (10)	
H31	0.598464	0.241717	0.270467	0.046*	
C32	0.63070 (13)	0.1393 (5)	0.3378 (3)	0.0428 (11)	
H32	0.634963	0.052331	0.307227	0.051*	
C33	0.64615 (12)	0.1545 (5)	0.4052 (3)	0.0405 (10)	
H33	0.661350	0.079018	0.420865	0.049*	
C34	0.63901 (9)	0.2812 (5)	0.4489 (2)	0.0294 (8)	
H34	0.649092	0.290304	0.495433	0.035*	

### Atomic displacement parameters (Å^2^)

**Table:**

	*U*^11^	*U*^22^	*U*^33^	*U*^12^	*U*^13^	*U*^23^
Ir1	0.01073 (8)	0.02118 (9)	0.01070 (8)	−0.00081 (5)	−0.00032 (5)	0.00108 (5)
P1	0.0238 (4)	0.0256 (5)	0.0237 (5)	0.0016 (4)	−0.0007 (4)	−0.0031 (4)
F1	0.0337 (13)	0.0576 (18)	0.0501 (15)	−0.0234 (13)	−0.0067 (12)	0.0149 (14)
F2	0.0249 (12)	0.086 (2)	0.0310 (13)	0.0105 (13)	0.0101 (10)	0.0011 (14)
F3	0.0420 (14)	0.0589 (18)	0.0361 (14)	0.0120 (13)	−0.0097 (11)	0.0143 (12)
F4	0.0491 (15)	0.0615 (18)	0.0211 (12)	−0.0119 (13)	−0.0074 (11)	−0.0070 (11)
F5	0.0402 (15)	0.0402 (16)	0.096 (3)	0.0011 (12)	−0.0179 (16)	−0.0321 (16)
F6	0.0400 (14)	0.0373 (15)	0.0687 (19)	−0.0080 (12)	−0.0104 (13)	−0.0112 (14)
F7	0.0417 (15)	0.0500 (16)	0.0534 (17)	0.0084 (13)	−0.0016 (13)	0.0212 (14)
F8	0.0387 (14)	0.072 (2)	0.0307 (13)	0.0182 (14)	0.0065 (11)	0.0179 (13)
F9	0.0390 (14)	0.0674 (19)	0.0368 (14)	0.0209 (14)	0.0073 (11)	−0.0052 (13)
F10	0.0391 (14)	0.0416 (14)	0.0368 (13)	0.0040 (11)	0.0145 (11)	0.0054 (11)
O1	0.0263 (13)	0.0339 (15)	0.0154 (11)	−0.0057 (11)	−0.0060 (10)	0.0043 (11)
O2	0.0170 (12)	0.0396 (16)	0.0261 (13)	−0.0069 (11)	0.0025 (10)	0.0079 (12)
N1	0.0156 (13)	0.0207 (15)	0.0153 (13)	0.0024 (11)	−0.0003 (10)	0.0040 (11)
N2	0.0149 (13)	0.0176 (14)	0.0134 (13)	−0.0014 (11)	0.0001 (10)	−0.0015 (11)
N3	0.0185 (14)	0.0307 (17)	0.0140 (13)	0.0078 (13)	−0.0012 (11)	−0.0010 (12)
N4	0.0185 (14)	0.0271 (16)	0.0165 (14)	−0.0024 (12)	0.0024 (11)	0.0002 (12)
C1	0.0147 (15)	0.0291 (19)	0.0172 (16)	−0.0017 (14)	0.0027 (13)	0.0030 (14)
C2	0.0197 (16)	0.0290 (19)	0.0157 (16)	0.0017 (14)	0.0036 (13)	0.0047 (14)
C3	0.0235 (17)	0.0158 (16)	0.0159 (16)	0.0016 (14)	−0.0025 (13)	−0.0011 (13)
C4	0.0168 (16)	0.0205 (17)	0.0185 (16)	−0.0011 (13)	−0.0012 (13)	0.0031 (13)
C5	0.0148 (15)	0.0172 (16)	0.0137 (15)	−0.0005 (12)	0.0001 (12)	−0.0009 (12)
C6	0.0132 (15)	0.0200 (17)	0.0144 (15)	0.0003 (13)	−0.0012 (12)	−0.0004 (13)
C7	0.0165 (16)	0.0227 (18)	0.0175 (16)	−0.0017 (14)	−0.0013 (12)	0.0009 (13)
C8	0.0167 (16)	0.0226 (18)	0.0221 (17)	−0.0034 (14)	0.0039 (13)	−0.0002 (14)
C9	0.0248 (17)	0.0250 (18)	0.0132 (15)	−0.0004 (15)	0.0022 (13)	0.0010 (14)
C10	0.0200 (16)	0.0214 (17)	0.0117 (14)	−0.0002 (13)	−0.0010 (12)	0.0007 (13)
C11	0.034 (2)	0.048 (3)	0.0237 (19)	−0.015 (2)	−0.0169 (17)	0.0066 (18)
C12	0.0179 (17)	0.043 (2)	0.031 (2)	−0.0074 (17)	0.0000 (15)	0.0066 (18)
C13	0.0138 (15)	0.042 (2)	0.0157 (16)	−0.0058 (15)	−0.0039 (13)	0.0127 (16)
C14	0.0242 (19)	0.041 (2)	0.0243 (19)	−0.0083 (18)	−0.0061 (15)	0.0091 (17)
C15	0.0239 (19)	0.052 (3)	0.0238 (19)	−0.0073 (19)	−0.0062 (15)	0.0124 (19)
C16	0.0218 (19)	0.066 (3)	0.027 (2)	−0.013 (2)	−0.0083 (16)	0.017 (2)
C17	0.0206 (19)	0.071 (3)	0.0166 (18)	0.006 (2)	0.0009 (15)	0.0076 (19)
C18	0.0169 (17)	0.052 (3)	0.0146 (16)	0.0046 (17)	0.0007 (13)	0.0059 (16)
C19	0.0250 (18)	0.045 (2)	0.0174 (17)	0.0115 (17)	0.0002 (14)	0.0010 (17)
C20	0.038 (2)	0.057 (3)	0.026 (2)	0.019 (2)	0.0083 (18)	0.002 (2)
C21	0.052 (3)	0.043 (3)	0.033 (2)	0.017 (2)	0.004 (2)	−0.009 (2)
C22	0.047 (2)	0.035 (2)	0.031 (2)	0.007 (2)	0.0002 (19)	−0.0048 (19)
C23	0.029 (2)	0.030 (2)	0.0235 (19)	0.0066 (16)	−0.0012 (15)	−0.0023 (15)
C24	0.0136 (15)	0.040 (2)	0.0135 (15)	−0.0064 (15)	0.0009 (12)	0.0065 (15)
C25	0.0188 (17)	0.043 (2)	0.0233 (18)	0.0023 (16)	−0.0021 (14)	0.0074 (17)
C26	0.0242 (19)	0.051 (3)	0.0252 (19)	−0.0012 (18)	0.0007 (15)	0.0110 (19)
C27	0.0242 (19)	0.057 (3)	0.0210 (19)	−0.0041 (19)	0.0000 (15)	0.0141 (19)
C28	0.030 (2)	0.052 (3)	0.0167 (17)	−0.0143 (19)	0.0003 (15)	−0.0034 (18)
C29	0.0225 (17)	0.044 (2)	0.0155 (16)	−0.0089 (17)	0.0016 (14)	0.0011 (16)
C30	0.032 (2)	0.032 (2)	0.0161 (17)	−0.0114 (17)	0.0019 (15)	−0.0029 (15)
C31	0.050 (3)	0.040 (3)	0.025 (2)	−0.008 (2)	0.0014 (18)	−0.0052 (19)
C32	0.063 (3)	0.028 (2)	0.037 (2)	0.000 (2)	0.006 (2)	−0.0135 (19)
C33	0.046 (3)	0.030 (2)	0.046 (3)	0.004 (2)	0.004 (2)	−0.008 (2)
C34	0.0288 (19)	0.027 (2)	0.032 (2)	−0.0034 (16)	0.0027 (16)	0.0017 (17)

### Geometric parameters (Å, º)

**Table:**

Ir1—C24	2.014 (3)	C10—H10	0.9500
Ir1—C13	2.017 (3)	C11—H11A	0.9800
Ir1—N4	2.035 (3)	C11—H11B	0.9800
Ir1—N3	2.042 (3)	C11—H11C	0.9800
Ir1—N1	2.128 (3)	C12—H12A	0.9800
Ir1—N2	2.136 (3)	C12—H12B	0.9800
P1—F6	1.579 (3)	C12—H12C	0.9800
P1—F5	1.585 (3)	C13—C14	1.382 (6)
P1—F8	1.594 (3)	C13—C18	1.424 (6)
P1—F9	1.599 (3)	C14—C15	1.393 (6)
P1—F7	1.601 (3)	C14—H14	0.9500
P1—F10	1.605 (3)	C15—C16	1.373 (7)
F1—C15	1.347 (5)	C16—C17	1.364 (7)
F2—C17	1.352 (5)	C16—H16	0.9500
F3—C26	1.347 (5)	C17—C18	1.398 (5)
F4—C28	1.360 (5)	C18—C19	1.461 (6)
O1—C3	1.337 (4)	C19—C20	1.404 (6)
O1—C11	1.446 (5)	C20—C21	1.381 (7)
O2—C8	1.340 (4)	C20—H20	0.9500
O2—C12	1.435 (5)	C21—C22	1.373 (7)
N1—C1	1.347 (4)	C21—H21	0.9500
N1—C5	1.350 (4)	C22—C23	1.380 (6)
N2—C10	1.347 (4)	C22—H22	0.9500
N2—C6	1.360 (4)	C23—H23	0.9500
N3—C23	1.333 (5)	C24—C25	1.377 (6)
N3—C19	1.385 (5)	C24—C29	1.435 (6)
N4—C34	1.344 (5)	C25—C26	1.397 (5)
N4—C30	1.366 (5)	C25—H25	0.9500
C1—C2	1.369 (5)	C26—C27	1.361 (7)
C1—H1	0.9500	C27—C28	1.374 (7)
C2—C3	1.401 (5)	C27—H27	0.9500
C2—H2	0.9500	C28—C29	1.393 (5)
C3—C4	1.387 (5)	C29—C30	1.464 (6)
C4—C5	1.386 (5)	C30—C31	1.403 (6)
C4—H4	0.9500	C31—C32	1.372 (7)
C5—C6	1.481 (4)	C31—H31	0.9500
C6—C7	1.389 (5)	C32—C33	1.393 (7)
C7—C8	1.392 (5)	C32—H32	0.9500
C7—H7	0.9500	C33—C34	1.383 (6)
C8—C9	1.396 (5)	C33—H33	0.9500
C9—C10	1.370 (5)	C34—H34	0.9500
C9—H9	0.9500		
			
C24—Ir1—C13	86.02 (13)	H11A—C11—H11B	109.5
C24—Ir1—N4	81.16 (14)	O1—C11—H11C	109.5
C13—Ir1—N4	93.90 (15)	H11A—C11—H11C	109.5
C24—Ir1—N3	94.85 (14)	H11B—C11—H11C	109.5
C13—Ir1—N3	81.09 (15)	O2—C12—H12A	109.5
N4—Ir1—N3	173.84 (12)	O2—C12—H12B	109.5
C24—Ir1—N1	177.11 (12)	H12A—C12—H12B	109.5
C13—Ir1—N1	96.57 (12)	O2—C12—H12C	109.5
N4—Ir1—N1	97.36 (12)	H12A—C12—H12C	109.5
N3—Ir1—N1	86.83 (11)	H12B—C12—H12C	109.5
C24—Ir1—N2	101.08 (12)	C14—C13—C18	119.0 (3)
C13—Ir1—N2	172.49 (12)	C14—C13—Ir1	127.4 (3)
N4—Ir1—N2	89.64 (11)	C18—C13—Ir1	113.6 (3)
N3—Ir1—N2	95.76 (11)	C13—C14—C15	119.6 (4)
N1—Ir1—N2	76.39 (10)	C13—C14—H14	120.2
F6—P1—F5	179.0 (2)	C15—C14—H14	120.2
F6—P1—F8	90.37 (17)	F1—C15—C16	118.9 (4)
F5—P1—F8	90.54 (18)	F1—C15—C14	118.5 (4)
F6—P1—F9	89.44 (17)	C16—C15—C14	122.6 (4)
F5—P1—F9	90.93 (17)	C17—C16—C15	117.5 (4)
F8—P1—F9	90.75 (14)	C17—C16—H16	121.2
F6—P1—F7	89.52 (17)	C15—C16—H16	121.2
F5—P1—F7	89.57 (18)	F2—C17—C16	116.8 (4)
F8—P1—F7	179.25 (17)	F2—C17—C18	120.3 (4)
F9—P1—F7	89.99 (15)	C16—C17—C18	123.0 (4)
F6—P1—F10	90.31 (15)	C17—C18—C13	118.3 (4)
F5—P1—F10	89.32 (16)	C17—C18—C19	125.6 (4)
F8—P1—F10	89.40 (14)	C13—C18—C19	116.1 (3)
F9—P1—F10	179.71 (17)	N3—C19—C20	119.1 (4)
F7—P1—F10	89.86 (15)	N3—C19—C18	113.4 (3)
C3—O1—C11	117.3 (3)	C20—C19—C18	127.5 (4)
C8—O2—C12	117.8 (3)	C21—C20—C19	119.6 (4)
C1—N1—C5	118.1 (3)	C21—C20—H20	120.2
C1—N1—Ir1	124.4 (2)	C19—C20—H20	120.2
C5—N1—Ir1	116.8 (2)	C22—C21—C20	120.4 (4)
C10—N2—C6	117.8 (3)	C22—C21—H21	119.8
C10—N2—Ir1	126.2 (2)	C20—C21—H21	119.8
C6—N2—Ir1	116.0 (2)	C21—C22—C23	118.1 (5)
C23—N3—C19	119.4 (3)	C21—C22—H22	120.9
C23—N3—Ir1	124.7 (2)	C23—C22—H22	120.9
C19—N3—Ir1	115.8 (3)	N3—C23—C22	123.2 (4)
C34—N4—C30	120.1 (3)	N3—C23—H23	118.4
C34—N4—Ir1	123.7 (3)	C22—C23—H23	118.4
C30—N4—Ir1	116.2 (3)	C25—C24—C29	119.2 (3)
N1—C1—C2	122.9 (3)	C25—C24—Ir1	128.3 (3)
N1—C1—H1	118.5	C29—C24—Ir1	112.4 (3)
C2—C1—H1	118.5	C24—C25—C26	119.4 (4)
C1—C2—C3	119.0 (3)	C24—C25—H25	120.3
C1—C2—H2	120.5	C26—C25—H25	120.3
C3—C2—H2	120.5	F3—C26—C27	118.4 (4)
O1—C3—C4	125.3 (3)	F3—C26—C25	118.7 (4)
O1—C3—C2	116.2 (3)	C27—C26—C25	122.9 (4)
C4—C3—C2	118.5 (3)	C26—C27—C28	117.7 (4)
C5—C4—C3	119.0 (3)	C26—C27—H27	121.1
C5—C4—H4	120.5	C28—C27—H27	121.1
C3—C4—H4	120.5	F4—C28—C27	118.0 (4)
N1—C5—C4	122.4 (3)	F4—C28—C29	119.1 (4)
N1—C5—C6	114.9 (3)	C27—C28—C29	122.9 (4)
C4—C5—C6	122.7 (3)	C28—C29—C24	117.8 (4)
N2—C6—C7	122.1 (3)	C28—C29—C30	126.2 (4)
N2—C6—C5	115.4 (3)	C24—C29—C30	115.9 (3)
C7—C6—C5	122.6 (3)	N4—C30—C31	119.5 (4)
C6—C7—C8	119.0 (3)	N4—C30—C29	113.3 (3)
C6—C7—H7	120.5	C31—C30—C29	127.1 (4)
C8—C7—H7	120.5	C32—C31—C30	120.4 (4)
O2—C8—C7	124.1 (3)	C32—C31—H31	119.8
O2—C8—C9	117.0 (3)	C30—C31—H31	119.8
C7—C8—C9	118.9 (3)	C31—C32—C33	119.2 (4)
C10—C9—C8	118.7 (3)	C31—C32—H32	120.4
C10—C9—H9	120.7	C33—C32—H32	120.4
C8—C9—H9	120.7	C34—C33—C32	118.9 (4)
N2—C10—C9	123.6 (3)	C34—C33—H33	120.5
N2—C10—H10	118.2	C32—C33—H33	120.5
C9—C10—H10	118.2	N4—C34—C33	121.9 (4)
O1—C11—H11A	109.5	N4—C34—H34	119.0
O1—C11—H11B	109.5	C33—C34—H34	119.0
			
C5—N1—C1—C2	0.5 (5)	C14—C13—C18—C19	178.4 (3)
Ir1—N1—C1—C2	−169.9 (3)	Ir1—C13—C18—C19	−0.1 (4)
N1—C1—C2—C3	1.4 (6)	C23—N3—C19—C20	1.8 (5)
C11—O1—C3—C4	−5.0 (5)	Ir1—N3—C19—C20	178.8 (3)
C11—O1—C3—C2	175.2 (3)	C23—N3—C19—C18	−177.3 (3)
C1—C2—C3—O1	177.0 (3)	Ir1—N3—C19—C18	−0.3 (4)
C1—C2—C3—C4	−2.9 (5)	C17—C18—C19—N3	178.7 (3)
O1—C3—C4—C5	−177.4 (3)	C13—C18—C19—N3	0.3 (5)
C2—C3—C4—C5	2.5 (5)	C17—C18—C19—C20	−0.3 (6)
C1—N1—C5—C4	−1.0 (5)	C13—C18—C19—C20	−178.7 (4)
Ir1—N1—C5—C4	170.2 (3)	N3—C19—C20—C21	−1.1 (6)
C1—N1—C5—C6	−179.3 (3)	C18—C19—C20—C21	177.9 (4)
Ir1—N1—C5—C6	−8.1 (4)	C19—C20—C21—C22	−1.6 (7)
C3—C4—C5—N1	−0.6 (5)	C20—C21—C22—C23	3.3 (7)
C3—C4—C5—C6	177.6 (3)	C19—N3—C23—C22	0.0 (6)
C10—N2—C6—C7	−0.4 (5)	Ir1—N3—C23—C22	−176.6 (3)
Ir1—N2—C6—C7	178.8 (3)	C21—C22—C23—N3	−2.6 (7)
C10—N2—C6—C5	179.1 (3)	C29—C24—C25—C26	1.6 (5)
Ir1—N2—C6—C5	−1.7 (4)	Ir1—C24—C25—C26	−174.2 (3)
N1—C5—C6—N2	6.4 (4)	C24—C25—C26—F3	179.4 (3)
C4—C5—C6—N2	−171.9 (3)	C24—C25—C26—C27	1.5 (6)
N1—C5—C6—C7	−174.1 (3)	F3—C26—C27—C28	−179.9 (3)
C4—C5—C6—C7	7.6 (5)	C25—C26—C27—C28	−1.9 (6)
N2—C6—C7—C8	1.4 (5)	C26—C27—C28—F4	179.2 (3)
C5—C6—C7—C8	−178.1 (3)	C26—C27—C28—C29	−0.9 (6)
C12—O2—C8—C7	6.2 (5)	F4—C28—C29—C24	−176.2 (3)
C12—O2—C8—C9	−173.8 (3)	C27—C28—C29—C24	3.9 (6)
C6—C7—C8—O2	178.8 (3)	F4—C28—C29—C30	7.2 (6)
C6—C7—C8—C9	−1.2 (5)	C27—C28—C29—C30	−172.8 (4)
O2—C8—C9—C10	−180.0 (3)	C25—C24—C29—C28	−4.2 (5)
C7—C8—C9—C10	0.1 (5)	Ir1—C24—C29—C28	172.3 (3)
C6—N2—C10—C9	−0.9 (5)	C25—C24—C29—C30	172.8 (3)
Ir1—N2—C10—C9	−180.0 (3)	Ir1—C24—C29—C30	−10.7 (4)
C8—C9—C10—N2	1.0 (5)	C34—N4—C30—C31	0.6 (5)
C18—C13—C14—C15	0.4 (5)	Ir1—N4—C30—C31	178.9 (3)
Ir1—C13—C14—C15	178.7 (3)	C34—N4—C30—C29	−176.0 (3)
C13—C14—C15—F1	179.1 (3)	Ir1—N4—C30—C29	2.3 (4)
C13—C14—C15—C16	−0.7 (6)	C28—C29—C30—N4	−177.7 (4)
F1—C15—C16—C17	−179.1 (3)	C24—C29—C30—N4	5.6 (5)
C14—C15—C16—C17	0.7 (6)	C28—C29—C30—C31	6.0 (7)
C15—C16—C17—F2	179.2 (3)	C24—C29—C30—C31	−170.7 (4)
C15—C16—C17—C18	−0.4 (6)	N4—C30—C31—C32	−1.3 (6)
F2—C17—C18—C13	−179.5 (3)	C29—C30—C31—C32	174.9 (4)
C16—C17—C18—C13	0.1 (6)	C30—C31—C32—C33	0.4 (7)
F2—C17—C18—C19	2.1 (6)	C31—C32—C33—C34	1.0 (7)
C16—C17—C18—C19	−178.3 (4)	C30—N4—C34—C33	0.9 (6)
C14—C13—C18—C17	−0.1 (5)	Ir1—N4—C34—C33	−177.3 (3)
Ir1—C13—C18—C17	−178.6 (3)	C32—C33—C34—N4	−1.7 (7)

### Hydrogen-bond geometry (Å, º)

**Table:**

*D*—H···*A*	*D*—H	H···*A*	*D*···*A*	*D*—H···*A*
C11—H11*A*···F7^i^	0.98	2.51	3.240 (6)	131
C11—H11*B*···O2^ii^	0.98	2.52	3.294 (5)	136
C20—H20···F2	0.95	2.25	2.866 (6)	122
C31—H31···F4	0.95	2.25	2.869 (6)	122
C34—H34···F8^iii^	0.95	2.55	3.412 (5)	150

Symmetry codes: (i) −*x*+3/2, *y*−1/2, *z*; (ii) −*x*+3/2, −*y*+3/2, *z*+1/2; (iii) *x*, *y*−1, *z*.
